# Estratégias de Revascularização em Doentes com Infarto Agudo do Miocárdio em Choque Cardiogênico – Resultados do Registo Português de Síndromes Coronárias Agudas

**DOI:** 10.36660/abc.20190739

**Published:** 2021-05-06

**Authors:** Sofia Alegria, Ana Marques, Ana Catarina Gomes, Ana Rita F. Pereira, Daniel Sebaiti, Gonçalo Morgado, Rita Calé, Cristina Martins, Adriana Belo, Inês Rangel, Hélder Pereira

**Affiliations:** 1 Hospital Garcia de Orta EPE Almada Portugal Hospital Garcia de Orta EPE, Almada - Portugal.; 2 Centro Nacional Coleção de Dados em Cardiologia Coimbra Portugal Centro Nacional Coleção de Dados em Cardiologia, Coimbra - Portugal.

**Keywords:** Infarto do Miocárdio, Choque Cardiogênico, Revascularização Miocárdica, Síndrome Coronária Aguda, Disfunção Ventricular Esquerda, Mortalidade, Biomarcadores, Hemorragia Intracraniana

## Abstract

**Fundamento:**

Em doentes com infarto agudo do miocárdio (IAM), choque cardiogênico (CC) e doença multivaso (DMV) persistem dúvidas sobre a intervenção nas artérias não responsáveis.

**Objetivos:**

1) caracterizar a amostra de doentes com IAM, CC e DMV incluídos no Registo Nacional Português de Síndromes Coronárias Agudas (RNSCA); 2) comparar os eventos associados a diferentes estratégias de revascularização; e 3) identificar preditores de mortalidade intra-hospitalar nesta amostra.

**Métodos:**

Estudo observacional retrospetivo de doentes com IAM, CC e DMV incluídos no RNSCA entre 2010 e 2018. Compararam-se duas estratégias de revascularização: completa durante o procedimento índice (grupo 1); e completa diferida ou incompleta durante o internamento (grupo 2-3). O endpoint primário foi a ocorrência de reinfarto ou morte intra-hospitalar. A significância estatística foi definida por um valor p < 0,05.

**Resultados:**

Identificaram-se 127 doentes com IAM, CC e DMV (18,1% no grupo 1 e 81,9% no grupo 2-3), com idade média de 70 ± 12 anos e 92,9% com IAM com supradesnivelamento do segmento ST. O *endpoint* primário ocorreu em 47,8% dos doentes do grupo 1 e em 37,5% do grupo 2-3 (p = 0,359). As taxas de mortalidade intra-hospitalar, reinfarto, acidente vascular cerebral e hemorragia *major* foram também semelhantes nos dois grupos. Os preditores de mortalidade intra-hospitalar nesta amostra foram a presença na admissão de disfunção ventricular esquerda (OR 16,8), bloqueio completo de ramo direito (OR 7,6) e anemia (OR 5,2), (p ≤ 0,02).

**Conclusões:**

Entre os doentes com IAM, CC e DMV, incluídos no RNSCA, não se verificou diferença significativa entre revascularização completa no evento índex e completa diferida ou incompleta durante o internamento, relativamente à ocorrência de morte intra-hospitalar ou reinfarto. (Arq Bras Cardiol. 2021; 116(5):867-876)

## Introdução

Nos doentes que se apresentam com infarto agudo do miocárdio (IAM) em choque cardiogênico (CC), a revascularização da artéria responsável associa-se a uma melhoria do prognóstico.^1^ No entanto uma proporção significativa destes doentes apresenta doença multivaso (DMV)^2^ o que levanta a questão sobre a indicação e *timing* para revascularização das artérias não responsáveis.

As recomendações da Sociedade Europeia de Cardiologia (ESC) sobre abordagem do IAM com supradesnivelamento do segmento ST (IAMCSST) publicadas em 2017 defendiam a revascularização imediata das artérias não responsáveis nos doentes em CC (recomendação classe IIa, nível de evidência C).^3^

No entanto, os resultados do ensaio clínico Culprit-Shock, publicado no mesmo ano, desafiaram esta recomendação.^4^ Este estudo incluiu 706 doentes com IAM, CC e DMV, aleatorizados para duas estratégias de revascularização percutânea: angioplastia da artéria responsável, com a opção de revascularização diferida dos restantes vasos durante o internamento, ou angioplastia multivaso imediata. Os resultados demonstraram que o *endpoint* combinado de morte ou lesão renal grave com necessidade de técnica de substituição renal foi significativamente mais baixo nos doentes submetidos inicialmente apenas a angioplastia da artéria responsável.^4^

Estes dados foram fundamentais na alteração das recomendações mais recentes. Assim, as recomendações da ESC sobre revascularização miocárdica publicadas em 2018 atribuem uma recomendação classe III a esta estratégia.^5^

Desta forma, os objetivos deste trabalho foram: 1) caracterizar a amostra de doentes com IAM, CC e DMV incluídos no Registo Nacional Português de Síndromes Coronárias Agudas (RNSCA); 2) comparar os eventos associados a diferentes estratégias de revascularização; e 3) identificar preditores de mortalidade intra-hospitalar nesta amostra.

## Métodos

Estudo observacional com análise retrospetiva de doentes admitidos com IAM, apresentação em CC (classe Killip-Kimball IV) e DMV, incluídos no RNSCA entre Outubro de 2010 e Janeiro de 2018.

Foram comparadas três estratégias de revascularização: revascularização completa durante o evento índice – grupo 1; revascularização completa diferida durante o internamento – grupo 2; e revascularização incompleta durante o internamento – grupo 3.

Para a definição de lesão coronária significativa foram utilizados critérios angiográficos, considerando-se como significativa uma lesão com estenose igual ou superior a 50%. Considerou-se revascularização completa quando todas as lesões coronárias significativas foram revascularizadas.

### Definição de IAM

O IAM foi definido de acordo com o documento de definição de variáveis do Registo Nacional de Síndromes Coronárias Agudas.6

O IAM com Supradesnivelamento do segmento ST foi definido pela presença de supradesnivelamento persistente (> 30 minutos) do segmento ST > 1mm (0,1mV) em duas ou mais derivações contíguas ou bloqueio completo de ramo esquerdo (BCRE) de novo, em contexto clínico sugestivo de isquemia miocárdica.

O IAM sem Supradesnivelamento do segmento ST foi definido pela ausência de supradesnivelamento persistente (< 30 minutos) do segmento ST associada a elevação de biomarcadores de necrose miocárdica (troponina ou CK- mb) em contexto clínico sugestivo de isquemia miocárdica.

### Definição dos endpoints

Definiu-se como *endpoint* primário combinado a ocorrência de reinfarto ou morte intra-hospitalar. Os *endpoints* foram definidos de acordo com o documento de definição de variáveis do Registo Nacional de Síndromes Coronárias Agudas.^6^ Reinfarto foi definido pela recorrência de dor torácica sugestiva de isquemia, após resolução do episódio de dor da admissão, com duração superior a 20 minutos, acompanhada de alterações eletrocardiográficas e de nova elevação dos biomarcadores de necrose miocárdica em relação ao valor prévio (elevação de CK-MB duas vezes o valor de referência ou > 50% do valor prévio; ou elevação > 20% do valor da Troponina I/T em relação ao valor prévio).

Acidente vascular cerebral (AVC) isquémico foi definido pela instalação de novo de défices neurológicos focais sem evidência de hemorragia na tomografia computorizada (TC) cerebral durante o internamento hospitalar, e o AVC hemorrágico pela instalação durante o internamento hospitalar de défices neurológicos focais de novo com evidência de hemorragia na TC cerebral. A definição de complicação mecânica de IAM incluiu ruptura de parede livre, ruptura do septo interventricular e insuficiência mitral aguda grave, por envolvimento dos músculos papilares.

Hemorragia *major* durante o internamento hospitalar foi definida de acordo com a classificação GUSTO (hemorragia intracraniana ou hemorragia com compromisso hemodinâmico requerendo intervenção).^7^

### Análise Estatística

A caracterização das variáveis contínuas foi feita recorrendo a média amostral e desvio-padrão, ou mediana e intervalo interquartil, conforme a presença ou não de normalidade na distribuição dos dados. A comparação das médias foi realizada recorrendo ao teste t-Student não pareado ou ao teste não paramétrico Mann-Whitney. A normalidade foi testada com o teste de Kolmogorov-Smirnov. As variáveis categóricas foram caracterizadas por meio de percentagens e as associações entre grupos foram analisadas pelo teste de X^2^ ou pelo teste de Fisher, conforme apropriado. Foi ajustado um modelo de regressão logística multivariada para identificação dos preditores independentes de mortalidade intra-hospitalar, com ajuste para variáveis demográficas, diagnóstico, localização do IAMCSST, fatores de risco, antecedentes, frequência cardíaca, pressão arterial, ritmo, morfologia dos complexos QRS, artérias coronárias com lesão, função ventricular esquerda, dados laboratoriais e medicação prévia e intra-hospitalar.

Para a análise estatística foi utilizado o software SPSS 19.0.0.2. Um valor de p < 0,05 foi considerado estatisticamente significativo.

## Resultados

### Caracterização da Amostra

**Figura 1:**
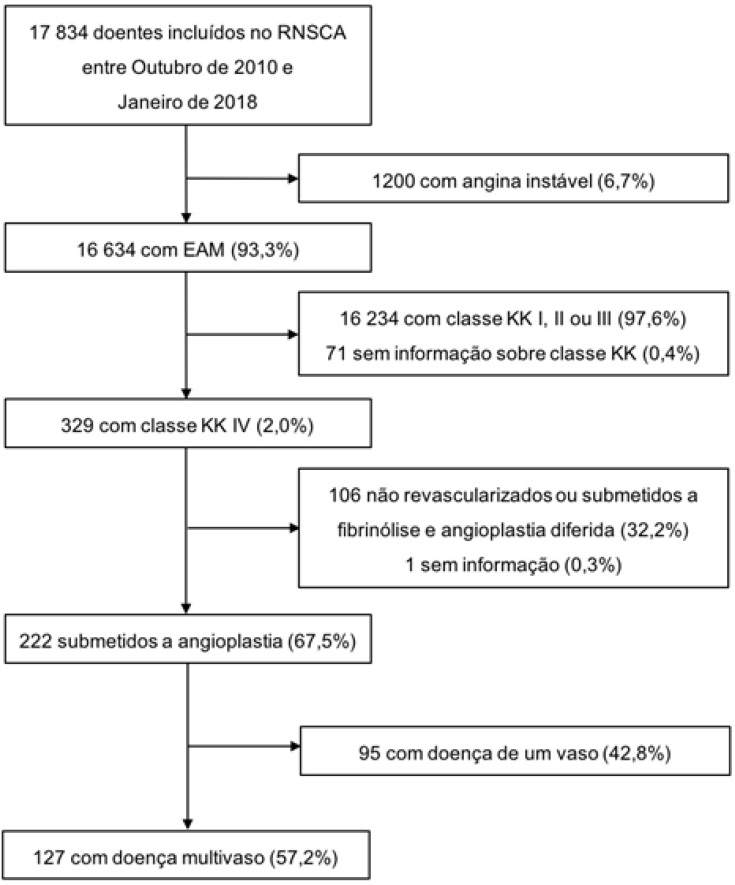
Fluxograma de inclusão de doentes na análise. IAM: infarto agudo do miocárdio; KK: Killip-Kimball; RNSCA: Registo Nacional de Síndromes Coronárias Agudas.

Entre os 17.834 doentes incluídos no RNSCA entre Outubro de 2010 e Janeiro de 2018, identificaram-se 222 doentes com IAM e CC na admissão, submetidos a angioplastia (1,2%) (Figura 1). Destes, 57,2% (n=127) apresentavam DMV tendo sido incluídos na análise (18,1% no grupo 1, n=23; 3,1% no grupo 2, n=4; 78,7% no grupo 3, n=100).

A caracterização da amostra encontra-se detalhada nas tabelas 1–4. Os doentes apresentavam uma idade média de 70 12 anos e predomínio do sexo masculino (68,5%, n = 87). Cerca de três quartos (72,5%) apresentavam história de hipertensão arterial, 33,1% de diabetes mellitus, 57,5% de dislipidemia, 23,0% de tabagismo, 14,5% de IAM e 8,2% de doença renal crónica; 4,2% apresentavam história familiar de doença coronária prematura.

**Tabela 1:** Características basais da amostra

	Amostra (n=127)	Grupo 1 (n=23)	Grupo 2-3 (n=104)	Valor p*
Idade (anos) – média ± DP	70 ± 12	63 ± 10	72 ± 12	< 0,001
Sexo masculino (%)	68,5	78,3	66,3	0,266
IMC (Kg/m2)	26,9 ± 4,2	28,2 ± 4,8	26,5 ± 4,0	0,081
Fumador (%)	23,0	45,5	18,0	0,006
HTA (%)	72,5	66,7	73,7	0,510
Diabetes *mellitus* (%)	33,1	27,3	34,3	0,504
Dislipidemia (%)	57,5	66,7	55,4	0,347
História familiar de doença coronária prematura (%)	4,2	10,5	2,6	0,174
IAM prévio (%)	14,5	13,6	14,7	1,000
ICP prévia (%)	10,3	13,0	9,7	0,704
Cirurgia de revascularização miocárdica prévia (%)	1,6	0,0	1,9	1,000
AIT/AVC prévio (%)	15,1	4,3	17,5	0,194
Doença vascular periférica (%)	5,7	4,3	6,0	1,000
DRC (%)	8,2	13,0	7,1	0,397

* Comparação entre revascularização completa e revascularização incompleta. AIT: acidente isquémico transitório; AVC: acidente vascular cerebral; DP: desvio padrão; DRC: doença renal crónica; IAM: infarto agudo do miocárdio; HTA: hipertensão arterial; ICP: intervenção coronária percutânea; IMC: índice de massa corporal.

**Tabela 2:** Características dos doentes na admissão/internamento hospitalar

	Amostra (n=127)	Grupo 1 (n=23)	Grupo 2-3 (n=104)	Valor p*
Transporte pela VMER (%)	32,4	52,9	28,4	0,048
Transporte por ambulância sem médico (%)	25,7	11,8	28,4	0,227
Transporte por meios próprios (%)	28,6	17,6	30,7	0,384
Transporte por outros meios (%)	13,3	17,7	12,5	0,462
Admissão em hospital sem laboratório de Hemodinâmica (%)	36,3	30,4	37,6	0,518
Tempo sintomas-admissão (minutos) – mediana (IIQ)	152 (82-270)	130 (90-223)	154 (79-271)	0,387
IAMCSST (%)	92,9	95,7	92,3	1,000
IAMSSST (%)	6,3	4,3	6,7	1,000
IAM indeterminado (%)	0,8	0,0	1,0	1,000
IAM de localização anterior (%)	50,8	72,7	45,8	0,023
IAM de localização inferior (%)	44,9	13,6	52,1	0,001
FC (bpm) – média ± DP	82 ± 33	93 ± 36	80 ± 32	0,162
PAS (mmHg) – média ± DP	93 ± 27	90 ± 27	94 ± 27	0,446
Fibrilhação auricular (%)	10,2	4,3	11,5	0,460
Creatinina (mg/dl) – mediana (IIQ)	1,2 (0,9-1,7)	1,5 (0,8-2,0)	1,2 (1,0-1,7)	0,835
Creatinina máxima (mg/dl) – mediana (IIQ)	1,6 (1,1-2,6)	1,6 (1,2-2,8)	1,6 (1,1-2,6)	0,731
Hemoglobina (g/dl) – média ± DP	13,3 ± 1,9	14,2 ± 1,8	13,1 ± 1,9	0,033
BNP (pg/mL) – mediana (IIQ)	388 (100-779)	88 (34-535)	456 (177-1235)	0,040
Função VE <40% (%)	61,0	77,8	57,3	0,107

* Comparação entre revascularização completa e revascularização incompleta. BNP: péptido natriurético cerebral (*brain natriuretic peptide*); IAM: infarto agudo do miocárdio; IAMCSST: infarto agudo do miocárdio com supradesnivelamento do segmento ST; IAMSSST: infarto agudo do miocárdio sem supradesnivelamento do segmento ST; FC: frequência cardíaca; IIQ: intervalo interquartis; PAS: pressão arterial sistólica; VE: ventrículo esquerdo; VMER: viatura médica de emergência e reanimação.

**Tabela 3:** Terapêutica / procedimentos intra-hospitalares

	Amostra (n=127)	Grupo 1 (n=23)	Grupo 2-3 (n=104)	valor p*
Aspirina (%)	96,1	91,3	97,1	0,222
Clopidogrel (%)	84,1	73,9	86,4	0,202
Ticagrelor (%)	16,8	23,5	15,4	0,476
Inibidores GP IIb-IIIa	37,6	52,2	34,3	0,110
Heparina não fraccionada (%)	66,7	65,2	67,0	0,870
Heparina de baixo peso molecular (%)	45,7	34,8	48,1	0,247
Bivalirudina (%)	0,8	0,0	1,0	1,000
Beta-bloqueante (%)	36,5	43,5	35,0	0,334
IECA (%)	46,5	34,8	49,0	0,215
ARA (%)	0,8	0,0	1,0	1,000
Antagonista da aldosterona (%)	21,3	30,4	19,2	0,263
Estatina (%)	74,0	73,9	74,0	0,990
Acesso vascular femoral (%)	66,4	60,9	67,6	0,534
Doença de 2 vasos (%)	58,7	100,0	51,1	< 0,001
Doença de 3 vasos (%)	41,3	0,0	48,9	< 0,001
Artéria responsável				
Tronco comum (%)	17,2	40,0	12,5	0,007
Descendente anterior (%)	25,9	40,0	22,9	0,112
Circunflexa (%)	10,3	5,0	11,5	0,688
Coronária direita (%)	35,3	5,0	41,7	0,002
Dispositivos de trombectomia (%)	39,3	36,4	40,0	0,752
Cateter de Swan-Ganz (%)	4,7	8,7	3,8	0,297
Balão intra-aórtico (%)	18,9	21,7	18,3	0,769
VMI (%)	37,0	43,5	35,6	0,478
VNI (%)	18,9	26,1	17,3	0,379
PM provisório (%)	21,3	8,7	24,0	0,158

* Comparação entre revascularização completa e revascularização incompleta. ARA: antagonista dos receptores da angiotensina; GP IIb-IIIa: glicoproteína IIb-IIIa; IECA: inibidores da enzima de conversão da angiotensina; N/D: não disponível; PM: pacemaker; VMI: ventilação mecânica invasiva; VNI: ventilação não invasiva.

**Tabela 4:** Eventos adversos durante o internamento

	Amostra (n=127)	Grupo 1 (n=23)	Grupo 2-3 (n=104)	valor p*
Reinfarto (%)	1,6	4,3	1,0	0,331
Complicação mecânica (%)	4,7	0,0	5,8	0,591
Bloqueio AV (%)	27,6	8,7	31,7	0,025
TV mantida (%)	9,4	8,7	9,6	1,000
Paragem cardiorrespiratória (%)	24,4	17,4	26,0	0,387
AVC (%)	0,8	0,0	1,0	1,000
Hemorragia major (%)	5,5	4,3	5,8	1,000
Morte intra-hospitalar (%)	37,8	43,5	36,5	0,535
Reinfarto ou morte intra-hospitalar (%)	39,4	47,8	37,5	0,359

* Comparação entre revascularização completa e revascularização incompleta. AV: auriculoventricular; AVC: acidente vascular cerebral; TV: taquicardia ventricular.

Cerca de um terço dos doentes (36,3%, n = 45) foram admitidos em centros sem Cardiologia de Intervenção e 28,6% (n = 30) recorreram ao serviço de urgência por meios próprios. A maioria apresentou diagnóstico de IAM com supradesnivelamento do segmento ST (IAMCSST) (92,9%), 6,3% IAM sem supradesnivelamento do segmento ST (IAMSSST) e 0,8% IAM de origem indeterminada. A artéria responsável foi o tronco comum em 17,2%, a descendente anterior em 25,9%, a circunflexa em 10,3% e a coronária direita em 35,3% dos casos. Utilizou-se balão intra-aórtico em 18,9% dos doentes (n = 24) e em nenhum doente foi implantado um dispositivo de assistência ventricular, enquanto 37,0% necessitaram de ventilação mecânica invasiva (n = 47). O *endpoint* primário ocorreu em 39,4% dos doentes (n = 50) e a taxa de mortalidade intra-hospitalar foi de 37,8% (n = 48).

### Comparação entre Estratégias de Revascularização

Considerando o pequeno número de doentes no grupo 2, a comparação das estratégias de revascularização foi realizada entre os grupos 1 e o grupo 2-3 (revascularização completa no procedimento índice vs. completa diferida ou incompleta durante o internamento), correspondendo a 18,1% dos doentes no grupo 1 (n=23) e 81,9% no grupo 2-3 (n=104).

Na comparação entre grupos constatou-se que os doentes do grupo 1 eram mais novos (63 ± 10 vs. 72 ± 12 anos, p < 0,001) e apresentavam maior prevalência de hábitos tabágicos (45,5 vs. 18,0%, p=0,006); na admissão tinham maior prevalência de ritmo sinusal (95,7 vs. 76,0%, p=0,043), valores de hemoglobina (Hg) mais altos (14,2 8 gr/dl vs. 13,1± 1,9 gr/dl, p=0,033) e apresentavam um valor de péptido natriurético cerebral (*brain natriuretic peptide* – BNP) mais baixo (mediana 88; intervalo interquartis (IIQ) 34-535 vs. 455,5; IIQ 176,5-1234,5 pg/ml), p=0,040 (Tabelas 1 e 2). O infarto anterior foi mais prevalentes no grupo 1 (72,7% vs. 45,8%, p= 0,023) e o infarto inferior no grupo 3 (13,6% vs. 52,1%, p=0,001) (Tabela 2). Relativamente à anatomia coronária constatou-se ainda que os doentes do grupo 1 apresentavam todos doença de dois vasos, pelo que a prevalência de doença de três vasos foi superior no grupo 2-3 (0,0% vs. 48,9%, p < 0,001). Por outro lado, o tronco comum foi mais frequente como artéria responsável no grupo 1 (40,0 vs. 12,5%; p = 0,007), enquanto a coronária direita foi mais frequente no grupo 3 (5,0 vs. 41,7%; p = 0,002) (Tabela 3). De referir ainda que não houve diferenças entre o valor de creatinina (Cr) máxima durante o internamento nos dois grupos (Tabela 2).

**Figura 2:**
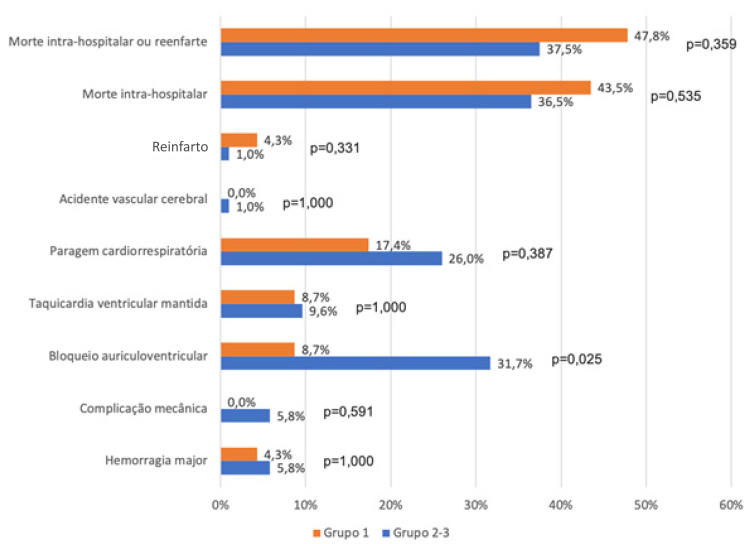
Fluxograma de inclusão de doentes na análise. IAM: infarto agudo do miocárdio; KK: Killip-Kimball; RNSCA: Registo Nacional de Síndromes Coronárias Agudas.

O *endpoint* primário ocorreu em 47,8% (n = 11) dos doentes do grupo 1 e em 37.5% (n = 39) do grupo 2-3 (p = 0,359). As taxas de mortalidade intra-hospitalar, re-infarto, AVC e hemorragia *major* (definida de acordo com os critérios GUSTO) também foram semelhantes entre os dois grupos, embora tenha havido maior incidência de bloqueio auriculoventricular de 2º grau Mobitz II ou de 3º grau no grupo 2-3 (8,7 vs. 31,7%; p = 0,025) (Tabela 4 e Figura 2).

### Preditores de Mortalidade Intra-Hospitalar

Na análise de regressão logística multivariada os preditores independentes de mortalidade intra-hospitalar foram a presença de disfunção ventricular esquerda (fração de ejeção inferior a 40%) (OR 16,79; IC 95% 5,03-56,02; p = 0,001), a presença de bloqueio completo de ramo direito (BCRD) (OR 7,60; IC 95% 2,22-25,97; p = 0,001) e hemoglobina inferior a 12 g/dl na admissão (OR 5,18; IC 95% 1,82-14,76; p = 0,002) (Tabela 5).

**Tabela 5:** Preditores independentes de mortalidade intra-hospitalar na amostra total na admissão

Preditores	Beta	OR	IC 95%	p-valor
Função VE < 40%	2,821	16,79	5,03-56,02	0,001
BCRD	2,028	7,60	2,22-25,97	0,001
Hb < 12 gr/dl	1,645	5,18	1,82-14,76	0,002

BCRD: bloqueio completo de ramo direito; Hb-hemoglobina; OR: odds ratio; VE: ventrículo esquerdo.

## Discussão

A DMV é frequente nos doentes com IAM e está relacionada com pior prognóstico, incluindo aumento de mortalidade.^8^ Este estudo, que incluiu uma amostra de doentes com IAM, CC na admissão e DMV, incluídos no RNSCA, mostra que, ao contrário do que defendiam as recomendações anteriores, a prática mais frequente era a realização de revascularização apenas da artéria responsável no procedimento índice.

Por outro lado, os resultados sugerem que a realização de revascularização completa no procedimento índice em comparação com revascularização diferida no mesmo internamento ou incompleta durante o internamento, não apresentou diferenças significativas relativamente ao *endpoint* combinado de reinfarto ou morte intra-hospitalar, pelo que esta estratégia parece ser segura.

Na comparação entre grupos constatou-se que os doentes submetidos a revascularização completa eram mais novos, na admissão estavam mais frequentemente em ritmo sinusal, apresentavam valores de Hg mais altos e um valor de BNP mais baixo, ou seja, eram doentes mais jovens, menos frágeis e provavelmente apresentavam quadros menos graves e, consequentemente, com menor risco para revascularização completa, nomeadamente em relação à ocorrência de nefropatia de contraste.

Nos doentes com IAM e DMV a angioplastia da lesão responsável é de facto o *standard of care*, mas a abordagem das restantes lesões tem sido um tema bastante controverso. Os resultados dos últimos ensaios clínicos aleatorizados, incluindo o PRAMI (*Preventative angioplasty in acute myocardial infarction*),^9^ o CvLPRIT (*Complete versus lesion-only primary PCI trial*),^10^ o DANAMI-3 PRIMULTI (*The third danish study of optimal acute treatment of patients with ST-segment elevation myocardial infarction: primary PCI in patients with ST-elevation myocardial infarction and multivessel disease*)^11^ e o COMPARE-ACUTE (*Comparison between FFR guided revascularization versus conventional strategy in acute STEMI patients with MVD*) trial,^12^ sugerem que a revascularização completa destes doentes poderá ser benéfica, contribuindo, por exemplo, para a recuperação da função ventricular e do estado hemodinâmico.^3^ Na realidade, o argumento para a revascularização completa baseia-se no potencial de melhorar a perfusão e função miocárdica global, embora a sua realização no evento índice coloque outros problemas adicionais, nomeadamente a indução de mais isquemia, sobrecarga de volume e agravamento da função renal associada ao aumento da dose de contraste utilizada.^8^ Salienta-se que em todos os estudos ocorreu redução do *endpoint* primário no grupo de revascularização completa, à custa sobretudo da redução da necessidade de revascularização adicional e da ocorrência de IAM não fatal, mas sem redução significativa da mortalidade.

Relativamente ao melhor *timing* de revascularização das artérias não responsáveis (imediata vs. diferida) não existe ainda evidência suficiente, sendo que nos ensaios foram utilizadas diferentes estratégias: revascularização das artérias não responsáveis no procedimento índice (PRAMI and Compare-Acute),^9^^,^^12^ revascularização diferida durante o internamento (DANAMI-3–PRIMULTI),^11^ ou em qualquer altura antes da alta (imediata ou diferida) (CVLPRIT).^10^

Neste contexto, em 2017, as recomendações de IAMCSST do ESC atualizaram as indicações sobre a estratégia de revascularização nos doentes com DMV, atribuindo uma recomendação classe II, nível de evidência A, à revascularização completa por rotina antes da alta hospitalar.^3^

No entanto, os doentes em CC não foram incluídos nestes últimos ensaios. Já o ensaio CULPRIT-SHOCK^4^ (*Culprit lesion only PCI versus multivessel PCI in cardiogenic shock*) demonstrou que, em doentes com IAM e CC, o tratamento por rotina das lesões não responsáveis durante a angioplastia primária se associou a um aumento do *endpoint* combinado de mortalidade e lesão renal aguda grave com necessidade de técnica de substituição renal. Com base nestes resultados, as últimas recomendações do ESC sobre revascularização miocárdica, publicadas em 2018, consideram que a revascularização das artérias não responsáveis durante a angioplastia primária nestes doentes não deve ser realizada, sendo uma recomendação de classe III.^5^

Tendo em conta a evidência mais recente é também da maior relevância avaliar os dados da vida real. No presente estudo, os doentes submetidos a revascularização completa no evento índex apresentaram maior taxa de mortalidade intra-hospitalar e do *endpoint* combinado de morte intra-hospitalar ou reinfarto, embora esta diferença não tenha atingido significado estatístico (43,5 vs. 36,5%, p=0,535; 47,8 vs. 37,5%, p=0,359, respetivamente). A ocorrência de reinfarto, AVC ou hemorragia *major* foram semelhantes entre os dois grupos. Comparativamente, o ensaio Culprit-Shock demonstrou superioridade com a revascularização apenas da artéria responsável (com a possibilidade de revascularização completa diferida) com redução do *endpoint* combinado de mortalidade a 30 dias ou lesão renal aguda grave com necessidade de técnica de substituição renal (43,3 vs. 51,6%; HR 0.84, IC 95% 0,72–0,98; p = 0,03) e da mortalidade a 30 dias.^4^ No RNSCA não existe informação sobre necessidade de técnica de substituição renal pelo que não foi possível analisar este evento, embora se tenha constatado que o valor de creatinina máxima durante o internamento não foi diferente na comparação dos grupos em estudo.

Existem ainda outros aspetos que carecem de melhor esclarecimento, nomeadamente no que diz respeito à identificação das lesões não responsáveis que beneficiam de revascularização (angiografia, avaliação funcional ou imagiologia intracoronária) e ao melhor *timing* da realização do procedimento diferido. Na realidade, nos principais ensaios aleatorizados a decisão de angioplastia dos vasos não responsáveis foi guiada de diferentes formas, nomeadamente por angiografia com decisão de intervir em lesões com estenose superior a 50% (PRAMI).^9^ ou superior a 70% (CvLPRIT),^10^ ou ainda por avaliação funcional guiada por *fractional flow reserve* (FFR) (DANAMI-3–PRIMULTI e Compare-Acute).^11^^,^^12^

Neste trabalho, o facto da amostra de doentes que realizou revascularização completa diferida durante o internamento ser pequena condicionou a comparação das estratégias, não sendo possível avaliar a presença de diferenças entre a revascularização completa no evento índice *versus* revascularização completa diferida. No ensaio Culprit-Shock efetivamente houve possibilidade de revascularização completa durante a angioplastia primária *versus* revascularização apenas da artéria responsável com a possibilidade de revascularização diferida das restantes artérias. Salienta-se, no entanto, que neste último grupo apenas foi realizada revascularização diferida durante o internamento em aproximadamente 18% dos doentes.^4^ De forma semelhante, diversas meta-análises incluindo estudos aleatorizados e não aleatorizados com doentes com IAMCSST com ou sem CC também demonstraram mortalidade semelhante ou superior com revascularização completa num único procedimento *versus* apenas da artéria responsável, mas redução da mortalidade a curto e longo prazo com revascularização completa diferida em comparação com as restantes estratégias.^13^^–^^15^

Os preditores de mortalidade intra-hospitalar nesta amostra, para além da disfunção ventricular esquerda que tem sido já extensamente descrita na literatura,^5^^,^^16^^,^^17^ foram a presença de BCRD e de anemia na admissão, em concordância com outros trabalhos já publicados. Em relação ao BCRD a sua prevalência no contexto de SCA é de cerca de 6 a 10% e tem sido associada ao aumento da mortalidade intra-hospitalar, sobretudo nos doentes com IAMCSST e com BCRD de novo. Esta associação será provavelmente justificada pela facto de a irrigação do ramo direito do feixe de His ser feita principalmente por ramos da artéria descendente anterior.^18^^–^^20^ Neste contexto, as últimas recomendações do ESC de IAMCSST sugerem que a estratégia de angioplastia primária deve ser considerada na presença de BCRD e isquemia persistente.^4^ Relativamente à anemia, estudos prévios têm demonstrado que a sua presença se associa a pior prognóstico nos doentes com IAM, nomeadamente nos doentes em CC, verificando-se maior ocorrência de hemorragia *major* e mortalidade a curto e longo prazo.^21^^,^^22^

Outro aspeto particularmente interessante na análise deste registo, e que nos deve fazer refletir, é a elevada percentagem de doentes (cerca de um terço) que recorre ao hospital por meios próprios o que poderá ter um impacto no tempo até revascularização condicionando o prognóstico. Estes dados reforçam a ideia que é fundamental otimizar a via verde coronária, atuando sobretudo no tempo entre os sintomas e o primeiro contacto médico de forma a obter redução na mortalidade em geral e particularmente nestes doentes mais graves que apresentam elevada mortalidade. Outro ponto que importa ressalvar é que apesar dos avanços na terapêutica de revascularização se terem associado a melhorias na sobrevida nestes doentes, ainda persistem disparidades regionais e a mortalidade intra-hospitalar mantém-se elevada (37,8%), embora em concordância com a literatura publicada (27-51%).^23^

### Limitações

As principais limitações deste estudo estão relacionadas com o facto de se tratar de um estudo observacional, incluindo o viés de seleção nas estratégias utilizadas e os fatores de confundimento não quantificados relacionados com os *outcomes*. Este aspeto poderá ser particularmente relevante nos doentes incluídos no grupo de revascularização incompleta, não se podendo excluir que alguns destes doentes tenham falecido antes de ter sido realizada nova intervenção, em detrimento de a estratégia ter sido selecionada com base em critérios clínicos. Outra questão relevante é a ausência de um critério uniforme para a tomada de decisão sobre revascularização das artérias não responsáveis, nomeadamente uma percentagem de estenose na angiografia ou a necessidade de avaliação funcional ou imagiológica intracoronária, no entanto esta prática reflete o que se verifica na vida real. Finalmente, este estudo avalia a estratégia de revascularização completa no procedimento índice *versus* revascularização completa diferida ou incompleta, mas não apresentava um número suficiente de doentes no grupo submetido a revascularização completa diferida, que nos permita avaliar o melhor *timing* de revascularização das lesões não responsáveis.

## Conclusões

Nesta amostra de doentes com IAM, CC na admissão e DMV, incluídos no RNSCA, a realização de revascularização completa no procedimento índice em comparação com revascularização completa diferida no mesmo internamento ou incompleta durante o internamento não apresentou diferença significativa relativamente ao *endpoint* combinado de reinfarto ou morte intra-hospitalar.
