# Supplementary material for: Estratégias de Revascularização em Doentes com Infarto Agudo do Miocárdio em Choque Cardiogênico – Resultados do Registo Português de Síndromes Coronárias Agudas
Source: Arq Bras Cardiol. 2021 May 6;116(5):867–76. [Article in Portuguese] doi: 10.36660/abc.20190739 (PMC8121485; doi:10.36660/abc.20190739)
Supplement: Translation

# Translation: Revascularization Strategies in Patients with Acute Myocardial Infarction and Cardiogenic Shock: Results from the Portuguese Registry on Acute Coronary Syndromes

**Authors:** Sofia Alegria, Ana Marques, Ana Catarina Gomes, Ana Rita F. Pereira, Daniel Sebaiti, Gonçalo Morgado, Rita Calé, Cristina Martins, Adriana Belo, Inês Rangel, Hélder Pereira

**Affiliations:** 1 Almada Portugal Hospital Garcia de Orta EPE, Almada - Portugal.; 2 Coimbra Portugal Centro Nacional Coleção de Dados em Cardiologia, Coimbra - Portugal.

**Keywords:** Myocardial Infarction, Shock, Cardiogenic, Myocardial Revascularization, Acute Coronary Syndrome, Ventricular Dysfunction, Left, Mortality, Biomarkers, Intracranial Hemorrhage

## Abstract

**Background:**

In patients with acute myocardial infarction (MI), cardiogenic shock (CS), and multivessel disease (MVD) questions remain unanswered when it comes to intervention on non-culprit arteries.

**Objective:**

This article aims to 1) characterize patients with MI, CS and MVD included in the Portuguese Registry on Acute Coronary Syndromes (ProACS); 2) compare different revascularization strategies in the sample; 3) identify predictors of in-hospital mortality among these patients.

**Methods:**

Observational retrospective study of patients with MI, CS and MVD included in the ProACS between 2010 and 2018. Two revascularization strategies were compared: complete during the index procedure (group 1); and complete or incomplete during the index hospitalization (groups 2-3). The primary endpoint was a composite of in-hospital death or MI. Statistical significance was defined by a p-value <0.05.

**Results:**

We identified 127 patients with MI, CS, and MVD (18.1% in group 1, and 81.9% in groups 2-3), with a mean age of 7012 years, and 92.9% of the sample being diagnosed with ST-segment elevation MI (STEMI). The primary endpoint occurred in 47.8% of the patients in group 1 and 37.5% in group 2-3 (p = 0.359). The rates of in-hospital death, recurrent MI, stroke, and major bleeding were also similar. The predictors of in-hospital death in this sample were the presence of left ventricle systolic dysfunction on admission (OR 16.8), right bundle branch block (OR 7.6), and anemia (OR 5.2) (p ≤ 0.02 for both).

**Conclusions:**

Among patients with MI, CS, and MVD included in the ProACS, there was no significant difference between complete and incomplete revascularization during the index hospitalization regarding the occurrence of in-hospital death or MI. (Arq Bras Cardiol. 2021; 116(5):867-876)

## Introduction

In patients with acute myocardial infarction (MI) and cardiogenic shock (CS), revascularization of the culprit artery is associated with improved prognosis.^1^ Even so, a significant number of these patients evolve with multivessel disease (MVD),^2^ which raises doubts regarding the indication and timing of the revascularization of non-culprit arteries.

The 2017 European Society of Cardiology (ESC) guidelines for the management of ST-segment elevation MI (STEMI) recommend immediate revascularization of non-culprit arteries in patients with CS (class IIa recommendation, level of evidence C).^3^

However, the results of the CULPRIT-SHOCK clinical trial, published in the same year, challenged this recommendation.^4^ This trial included 706 patients with acute MI, CS and MVD randomly assigned to one of two initial revascularization strategies: percutaneous coronary intervention (PCI) of the culprit lesion only, with the option of staged revascularization of remaining lesions, or immediate multivessel PCI. The results showed that the primary endpoint—a composite of death or severe renal failure leading to renal-replacement therapy within 30 days after randomization—was significantly lower among patients submitted to PCI of the culprit lesion only.^4^

Considering these results, the most recent 2018 ESC guidelines on myocardial revascularization attributes a class III recommendation to routine revascularization of non-culprit lesions during primary PCI in patients with STEMI and CS.^5^

In this regard, the aims of the current study were: 1) to characterize the sample of patients with acute MI, CS and MVD included in the Portuguese Registry on Acute Coronary Syndromes (ProACS); 2) to compare outcomes associated with different revascularization strategies; and 3) to identify predictors of in-hospital mortality.

## Methods

Retrospective observational study of patients admitted with acute MI, presenting with CS (Killip-Kimball class IV) and MVD, included in the ProACS between October 2010 and January 2018.

Three revascularization strategies were compared: complete revascularization during the index procedure (group 1); complete staged revascularization during hospitalization (group 2); and incomplete revascularization during hospitalization (group 3).

The definition of a significant coronary lesion was based on angiographic criteria, that is, a lesion associated with stenosis of at least 50%. Complete revascularization was defined as revascularization of all significant lesions.

### Definition of Acute MI

Acute MI was defined according to the definitions of variables in the ProACS.^6^ Therefore, STEMI was described as the presence of persistent (lasting more than 30 minutes) ST-segment elevation above 1 mm (0.1 mV) in at least two contiguous leads or de novo left bundle branch block (LBBB), in a clinical setting suggestive of myocardial ischemia, while non-ST-segment elevation MI (NSTEMI) was defined by the absence of persistent ST-segment elevation associated with elevation of biomarkers of myocardial necrosis (troponin or CK-mb) in a clinical setting suggestive of myocardial ischemia.

### Endpoints

The primary endpoint was a composite of in-hospital death or reinfarction. The endpoints were defined according to the definitions of variables in the ProACS.^6^ Reinfarction was defined by the recurrence of chest pain suggestive of ischemia after resolution of the admission pain episode, lasting more than 20 minutes, with concomitant electrocardiographic changes and new elevation of biomarkers of myocardial necrosis in comparison with the previous level (elevation of CK-mb of at least twice the reference level or at least 50% more than the previous level; or elevation of I or T troponin of at least 20% more than the previous level).

Ischemic stroke was defined by the occurrence of de *novo* focal neurological deficits without evidence of hemorrhage in head computed tomography (CT) scan during hospitalization, as well as hemorrhagic stroke by the occurrence of de *novo* focal neurological deficits with concomitant hemorrhage in head CT scan. The definition of mechanical complication of MI includes left ventricular free-wall rupture (LVFWR), ventricular septal rupture and severe acute mitral regurgitation due to involvement of the papillary muscles.

Major bleeding during hospitalization was defined according to the criteria by the Global Strategies for Opening Occluded Coronary Arteries (GUSTO): intracranial bleeding or bleeding with hemodynamic compromise requiring intervention.^7^

### Statistical Analysis

Continuous variables were expressed by mean and standard deviation or median and interquartile range, according to the analysis of normality in the distribution of data, assessed with the Kolmogorov-Smirnov test. These variables were compared using the unpaired t-Student test or the non-parametric Mann-Whitney test. Categorical variables were shown as percentages, and the association between groups was assessed by the Chi-square or the Fisher test, as appropriate. The multivariate logistic regression was used to identify independent predictors of in-hospital mortality, with adjustment for demographical variables, diagnosis, STEMI location, cardiovascular risk factors, previous diagnosis, heart rate, blood pressure, heart rhythm, QRS complex morphology, coronary arteries with significant lesions, left ventricle ejection fraction (LVEF), laboratory data, and previous and in-hospital medication.

Statistical analysis was performed aided by the SPSS software, version 19.0.0.2. A p-value below 0.05 was considered statistically significant.

## Results

### Patient Characterization

Among the 17.834 patients included in the ProACS between October 2010 and January 2018, 222 patients with acute MI and CS on admission, submitted to PCI, were identified (1.2%) (Figure 1). Of these, 57.2% (n=127) presented with MVD and were included in the analysis (18.1% in group 1, n=23; 3.1% in group 2, n=4; 78.7% in group 3, n=100).

**Figure 1:** Flowchart of patient inclusion in the analysis. KK: Killip-Kimball; PCI: percutaneous coronary intervention; ProACS: Portuguese Registry on Acute Coronary Syndromes.

The characterization is detailed in Tables 1–4. Patients’ mean age was 70 ± 12 years and 68.5% of them (n=87) were males. About three-quarters of them (72.5%) had a history of arterial hypertension, 33.1% diabetes mellitus, 57.5% dyslipidemia, 23.0% smoking habits, 14.5% acute MI and 8.2% chronic kidney disease; 4.2% had a family history of premature coronary artery disease.

**Table 1:** Baseline characteristics

	Sample (n=127)	Group 1 (n=23)	Groups 2-3 (n=104)	p-value*
Age (years) – mean ± SD	70 ± 12	63 ± 10	72 ± 12	< 0.001
Male sex (%)	68.5	78.3	66.3	0.266
BMI (Kg/m2)	26.9 ± 4.2	28.2 ± 4.8	26.5 ± 4.0	0.081
Smoking habits (%)	23.0	45.5	18.0	0.006
Arterial hypertension (%)	72.5	66.7	73.7	0.510
Diabetes *mellitus* (%)	33.1	27.3	34.3	0.504
Dyslipidemia (%)	57.5	66.7	55.4	0.347
Family history of premature coronary artery disease (%)	4.2	10.5	2.6	0.174
Previous acute MI (%)	14.5	13.6	14.7	1.000
Previous PCI (%)	10.3	13.0	9.7	0.704
Previous CABG (%)	1.6	0.0	1.9	1.000
Previous TIA/stroke (%)	15.1	4.3	17.5	0.194
Peripheral artery disease (%)	5.7	4.3	6.0	1.000
Chronic kidney disease (%)	8.2	13.0	7.1	0.397

* Comparison between complete revascularization in the index procedure and complete staged or incomplete revascularization. BMI: body mass index; CABG: coronary artery bypass graft; MI: myocardial infarction; PCI: percutaneous coronary intervention; SD: standard deviation; TIA: transient ischemic attack.

**Table 2:** Patients’ characteristics on admission/hospitalization

	Sample (n=127)	Group 1 (n=23)	Groups 2-3 (n=104)	p-value *
Transportation by ambulance with physician (%)	32.4	52.9	28.4	0.048
Transportation by ambulance without physician (%)	25.7	11.8	28.4	0.227
Transportation by own means (%)	28.6	17.6	30.7	0.384
Transportation by other means (%)	13.3	17.7	12.5	0.462
Admission to primary PCI center (%)	36.3	30.4	37.6	0.518
Symptom-to-door time (minutes) – median (IQR)	152 (82-270)	130 (90-223)	154 (79-271)	0.387
STEMI (%)	92.9	95.7	92.3	1.000
NSTEMI (%)	6.3	4.3	6.7	1.000
MI with LBBB or ventricular paced rhythm (%)	0.8	0.0	1.0	1.000
Anterior MI (%)	50.8	72.7	45.8	0.023
Inferior MI (%)	44.9	13.6	52.1	0.001
Heart rate (bpm) – mean ± SD	82 ± 33	93 ± 36	80 ± 32	0.162
Systolic BP (mmHg) – mean ± SD	93 ± 27	90 ± 27	94 ± 27	0.446
Atrial fibrillation (%)	10.2	4.3	11.5	0.460
Creatinine on admission (mg/dl) – median (IQR)	1.2 (0.9-1.7)	1.5 (0.8-2.0)	1.2 (1.0-1.7)	0.835
Maximum creatinine (mg/dl) – median (IQR)	1.6 (1.1-2.6)	1.6 (1.2-2.8)	1.6 (1.1-2.6)	0.731
Hemoglobin (g/dl) – mean ± SD	13.3 ± 1.9	14.2 ± 1.8	13.1 ± 1.9	0.033
BNP (pg/mL) – median (IQR)	388 (100-779)	88 (34-535)	456 (177-1235)	0.040
LVEF <40% (%)	61.0	77.8	57.3	0.107

* Comparison between complete revascularization in the index procedure and complete staged or incomplete revascularization. BNP: brain natriuretic peptide; BP: blood pressure; bpm: beats per minute; IQR: interquartile range; LBBB: left bundle branch block; LVEF: left ventricular ejection fraction; MI: myocardial infarction; NSTEMI: non-ST-segment elevation myocardial infarction; PCI: percutaneous coronary intervention; SD: standard deviation; STEMI: ST-segment elevation myocardial infarction.

**Table 3:** In-hospital therapy and procedures

	Sample (n=127)	Group 1 (n=23)	Groups 2-3 (n=104)	p-value*
Aspirin (%)	96.1	91.3	97.1	0.222
Clopidogrel (%)	84.1	73.9	86.4	0.202
Ticagrelor (%)	16.8	23.5	15.4	0.476
GP IIb/IIIa inhibitors (%)	37.6	52.2	34.3	0.110
Unfractionated heparin (%)	66.7	65.2	67.0	0.870
Low-molecular-weight heparin (%)	45.7	34.8	48.1	0.247
Bivalirudin (%)	0.8	0.0	1.0	1.000
Beta-blocker (%)	36.5	43.5	35.0	0.334
ACEI (%)	46.5	34.8	49.0	0.215
ARB (%)	0.8	0.0	1.0	1.000
MRA (%)	21.3	30.4	19.2	0.263
Statin (%)	74.0	73.9	74.0	0.990
Femoral arterial access (%)	66.4	60.9	67.6	0.534
2-vessel coronary artery disease (%)	58.7	100.0	51.1	< 0.001
3-vessel coronary artery disease (%)	41.3	0.0	48.9	< 0.001
Culprit artery				
Left main (%)	17.2	40.0	12.5	0.007
Left anterior descending (%)	25.9	40.0	22.9	0.112
Left circumflex (%)	10.3	5.0	11.5	0.688
Right coronary artery (%)	35.3	5.0	41.7	0.002
Thrombectomy devices (%)	39.3	36.4	40.0	0.752
Swan-Ganz catheter (%)	4.7	8.7	3.8	0.297
Intra-aortic balloon pump (%)	18.9	21.7	18.3	0.769
IMV (%)	37.0	43.5	35.6	0.478
NIV (%)	18.9	26.1	17.3	0.379
Temporary pacemaker (%)	21.3	8.7	24.0	0.158

* Comparison between complete revascularization in the index procedure and complete staged or incomplete revascularization. ACEI: angiotensin-converting enzyme inhibitor; ARB: angiotensin II receptor blocker; GP IIb/IIIa: glycoprotein IIb/IIIa; IMV: invasive mechanical ventilation; MRA: mineralocorticoid receptor antagonist; NIV: non-invasive ventilation.

**Table 4:** Adverse events during hospitalization

	Sample (n=127)	Group 1 (n=23)	Groups 2-3 (n=104)	p-value*
Reinfarction (%)	1.6	4.3	1.0	0.331
Mechanical complication (%)	4.7	0.0	5.8	0.591
AV block (%)	27.6	8.7	31.7	0.025
Sustained VT (%)	9.4	8.7	9.6	1.000
Cardiac arrest (%)	24.4	17.4	26.0	0.387
Stroke (%)	0.8	0.0	1.0	1.000
Major bleeding (%)	5.5	4.3	5.8	1.000
In-hospital death (%)	37.8	43.5	36.5	0.535
In-hospital death or reinfarction (%)	39.4	47.8	37.5	0.359

* Comparison between complete revascularization in the index procedure and complete staged or incomplete revascularization. AV: atrioventricular; VT: ventricular tachycardia.

About one-third of patients (36.3%, n=45) was admitted to hospitals without on-site Interventional Cardiology and 28.6% (n=30) resorted to the emergency department by their own means. Most patients presented with STEMI (92.9%), 6.3% with NSTEMI and 0.8% with acute MI with LBBB or ventricular paced rhythm. The culprit artery was the left main artery (LM) in 17.2% of patients, the left anterior descending (LAD) in 25.9%, the left circumflex in 10.3%, and the right coronary artery (RCA) in 35.3%. The intra-aortic balloon pump (IABP) was used in 18.9% of patients (n=24) and none of them received a ventricular assist device (VAD), while 37.0% required invasive mechanical ventilation (n=47).

The primary endpoint occurred in 39.4% of patients (n=50), with in-hospital mortality rate of 37.8% (n=48).

### Comparison between Revascularization Strategies

Considering the small number of patients in group 2, the comparison between revascularization strategies was performed between group 1 and groups 2-3 as one (complete revascularization in the index procedure versus complete staged or incomplete revascularization during hospitalization), with 18.1% of patients in group 1 (n=23) and 81.9% in groups 2-3 (n=104). Most patients in groups 2-3 were revascularized by PCI, and only three patients (2.9%) were accepted for surgical revascularization of non-culprit arteries.

Patients in group 1 were younger (63±10 vs. 72±12 years, p<0.001) and had a higher prevalence of smoking habit (45.5 vs. 18.0%, p=0.006); on admission, they were more likely to be in sinus rhythm (95.7 vs. 76.0%, p=0.043), have a higher hemoglobin level (14.2±8.0 vs. 13.1±1.9 g/dl, p=0.033) and lower brain natriuretic peptide (BNP) levels (median 88, interquartile range (IQR) 34-535 vs. 455.5, IQR 176.5-1234.5 pg/ml, p=0.040) (Tables 1 and 2). There were no significant differences in peak serum creatinine (Cr) level during hospitalization between the two groups. Anterior MI was more common in group 1 (72.7 vs. 45.8%, p=0.023) and inferior MI in groups 2-3 (13.6 vs. 52.1%, p=0.001) (Table 2). Regarding coronary anatomy, all patients in group 1 had two-vessel disease, so three-vessel disease was more common in groups 2-3 (0.0 vs. 48.9%, p<0.001). The LM artery was frequently identified as culprit artery in group 1 (40.0 vs. 12.5%, p=0.007), while the RCA was more common in groups 2-3 (5.0 vs. 41.7%, p=0.002) (Table 3).

The primary endpoint occurred in 47.8% (n=11) of patients in group 1 and 37.5% (n=39) in groups 2-3 (p=0.359). The rates of in-hospital mortality, reinfarction, stroke, and GUSTO major bleeding did not differ significantly either between groups, although there was a higher incidence of Mobitz II second-degree or third-degree atrioventricular block in groups 2-3 (8.7 vs. 31.7%, p=0.025) (Table 4 and Figure 2).

**Figure 2:** Comparison of adverse events between the two revascularization strategies.

### Predictors of In-hospital Mortality

In multivariate logistic regression analysis, the independent predictors of in-hospital mortality were: presence of LV systolic dysfunction (ejection fraction below 40%) (OR 16.79; 95%CI 5.03-56.02; p=0.001), RBBB (OR 7.60; 95% CI 2.22-25.97; p=0.001), and hemoglobin below 12 g/dl on admission (OR 5.18; 95% CI 1.82-14.76; p=0.002) (Table 5).

**Table 5:** Independent predictors of in-hospital mortality in the overall sample on admission

Predictor	Beta	OR	95% CI	p-value
LVEF < 40%	2.821	16.79	5.03-56.02	0.001
RBBB	2.028	7.60	2.22-25.97	0.001
Hemoglobin < 12 g/dl	1.645	5.18	1.82-14.76	0.002

CI: confidence interval; LVEF: left ventricular ejection fraction; OR: odds ratio; RBBB: right bundle branch block.

## Discussion

MVD is common in patients with acute MI and is related to a worse prognosis, including increased mortality.^8^ This study included a sample of patients with MI, CS and MVD on admission, included in the ProACS, and shows that, contrary to previous recommendations, the most common practice in the index procedure was to perform PCI of the culprit lesion only.

Furthermore, we found no significant differences between complete revascularization in the index procedure compared with complete staged revascularization or incomplete revascularization during hospitalization, regarding the composite endpoint of in-hospital death or reinfarction, so this strategy seems safe in these patients.

In the comparison between groups, patients submitted to complete revascularization were found to be younger, more likely to be in sinus rhythm on admission, and presented higher levels of hemoglobin and lower levels of BNP. Being younger, they were less fragile and probably presented with a less severe condition, therefore, they were at lower risk for a complete revascularization, especially regarding the occurrence of contrast nephropathy.

In patients with acute MI and MVD, PCI of the culprit lesion is the standard care, but the management of the remaining lesions have been subject of controversy. The results of the most recent clinical trials, including the Preventive angioplasty in acute myocardial infarction (PRAMI),^9^ the Complete versus lesion-only primary PCI trial (CvLPRIT),^10^ the Third danish study of optimal acute treatment of patients with STEMI: primary PCI in patients with ST-elevation MI and MVD (DANAMI-3 PRIMULTI)^11^ and the Compare-Acute trial (Comparison between FFR guided revascularization versus conventional strategy in acute STEMI patients with MVD),^12^ suggest that complete revascularization may be beneficial in these patients, contributing, for instance, for the recovery of LV systolic function and the hemodynamic status.^3^ Actually, the argument for complete revascularization is based on the potential to improve myocardial perfusion and the global function, even though performing it in the index procedure raises additional issues, such as inducing further ischemia, volume overload and worsening of kidney function associated with the use of more contrast.^8^ It is important to note that in every trial the primary endpoint was lower in the group of complete revascularization, mostly due to a reduction in the need for additional revascularization and non-fatal MI, but without significant reduction in mortality.

There is not sufficient evidence regarding the best timing of revascularization of non-culprit arteries (immediate vs. deferred), since the trials used different strategies: revascularization in the index procedure (PRAMI and Compare-Acute),^9^^,^^12^ during hospitalization (DANAMI-3 PRIMULTI)^11^ or at any time before hospital discharge (immediate or deferred) (CvLPRIT).^10^

In this setting, in 2017 the ESC guidelines on the management of STEMI updated the recommendations on the revascularization strategy in patients with MVD, attributing a class IIa recommendation, level of evidence A, for routine complete revascularization before hospital discharge.^3^

However, patients in CS were not included in these trials, but the Culprit-Shock trial (*Culprit lesion only PCI versus multivessel PCI in cardiogenic shock*)^4^ showed that, in patients with acute MI and CS, routine treatment of non-culprit lesions during primary PCI was associated with an increase of the composite endpoint of death or severe renal failure leading to renal replacement therapy. Based on these results, the most recent ESC guidelines on myocardial revascularization, published in 2018, considered that revascularization of non-culprit arteries should not be performed during primary PCI, giving this strategy a class III recommendation.^5^

Considering the most recent evidence, it is important to assess real-world data. In the present study, patients submitted to complete revascularization in the index procedure presented higher rates of in-hospital mortality and of the composite endpoint of in-hospital death or reinfarction, although these differences did not reach statistical significance (43.5 vs. 36.5%, p=0.535; and 47.8 vs. 37.5%, p=0.359, respectively). The rates of reinfarction, stroke or major bleeding were similar between the two groups. In comparison, the Culprit-Shock trial showed superiority of culprit lesion-only PCI (with possibility of complete staged revascularization), translated in reduction of the composite endpoint of death or severe renal failure leading to renal replacement therapy within 30 days (43.3. vs. 51.6%; HR 0.84, 95% CI 0.72-0.98; p=0.03) and 30-day mortality.^4^ There is no information on renal replacement therapy in the ProACS, so it was not possible to assess this event, although no differences were found in maximum creatinine level during hospitalization between the two groups.

Other questions still need to be clarified, namely regarding the identification of the non-culprit lesions that could benefit from revascularization (by angiography, functional assessment or intracoronary imaging) and the best timing for the performance of the staged procedure. Actually, in the main randomized clinical trials, the decision to perform PCI in non-culprit arteries was guided in different ways, specifically by angiography with a decision to treat lesions with a stenosis above 50% (PRAMI)^9^ or 70% (CvLPRIT),^10^ or by functional assessment with fractional flow reserve (FFR) (DANAMI-3 PRIMULTI and Compare-Acute).^11^^,^^12^

In the present study, the small number of patients with complete staged revascularization during hospitalization limited the comparison between strategies, as we were not able to evaluate the difference between complete revascularization in the index procedure and complete staged revascularization.

The Culprit-Shock trial compared complete revascularization during primary PCI with culprit artery-only revascularization, with possibility of staged revascularization of the remaining arteries. It is important to highlight, however, that staged revascularization during hospitalization was only performed in 18% of patients.^4^ Similarly, several meta-analyses including both randomized and non-randomized studies in patients with STEMI with or without CS also showed similar or superior mortality rates with complete revascularization in a single procedure when compared to revascularization of the culprit artery only, but a reduction in short and long-term mortality with complete staged revascularization in comparison with the other strategies.^13^^–^^15^

The predictors of in-hospital mortality in this sample—apart from LV systolic dysfunction, which has already been extensively described^5^^,^^16^^,^^17^—were the presence of RBBB and anemia on admission, similarly to other published studies. The prevalence of RBBB in the setting of acute coronary syndrome is about 6 to 10% and it has been associated with increased in-hospital mortality, mostly in patients with STEMI and *de novo* RBBB. This association is probably justified by the irrigation of the right branch of the His bundle by branches of the LAD artery.^18^^–^^20^ Given this, the most recent ESC guidelines on STEMI suggest that primary PCI should be considered in the presence of RBBB and persistent ischemia.^4^ Considering that previous studies on anemia have shown its association with a worse prognosis in patients with acute MI, especially when CS is present, with a higher rate of major bleeding and short and long-term mortality.^21^^,^^22^

This is another issue that should make us reflect is the high percentage of patients (about one-third) that resorts to the hospital by their own means. This may have an impact in the time to revascularization and represent worse prognosis. These data reinforce the notion that it is critical to optimize the coronary care network (in Portugal, named as “coronary green pathway”), acting mostly in the time frame from symptoms to first medical contact in order to achieve a reduction in overall mortality, especially in these critical patients with such a high mortality rate. It is worth highlighting that, despite advances in revascularization therapy that have been associated with improved survival rate among these patients, there are still regional disparities and in-hospital mortality remains high (37.8%), which is in line with published data (27-51%).^23^

### Limitations

The main limitations of this study are related to its design as an observational study, including selection bias in the strategies used and unquantified confounding factors that may correlate with outcomes. This may be particularly relevant for patients included in the incomplete revascularization group, since one cannot exclude that some of them died before a staged intervention, instead of the revascularization strategy being selected based on clinical criteria. Another important issue is the absence of a uniform criteria for the decision regarding revascularization of non-culprit arteries, namely a stenosis percentage in angiography or the need for intracoronary functional or imaging assessment, yet this reflects real life practice. Finally, this study compared a strategy of complete revascularization in the index procedure with staged complete or incomplete revascularization, but the number of patients in the group of complete staged revascularization was insufficient for an assessment of the best timing for revascularization of non-culprit arteries.

## Conclusions

In this sample of patients with acute MI, CS on admission, and MVD included in the ProACS, there was no significant difference between complete revascularization in the index procedure and staged complete or incomplete revascularization during hospitalization when it comes to the composite endpoint of in-hospital death or reinfarction.
